# COVID-19-Related Psychological and Psychosocial Distress Among Parents and Youth With Physical Illness: A Longitudinal Study

**DOI:** 10.3389/fpsyt.2021.761968

**Published:** 2021-10-28

**Authors:** Mark A. Ferro, Samantha B. Meyer, Jennifer Yessis, Shannon V. Reaume, Ellen Lipman, Jan Willem Gorter

**Affiliations:** ^1^School of Public Health Sciences, University of Waterloo, Waterloo, ON, Canada; ^2^Department of Psychiatry and Behavioural Neurosciences, McMaster University, Hamilton, ON, Canada; ^3^Department of Pediatric Rehabilitation Medicine, University Medical Center Utrecht, Utrecht, Netherlands

**Keywords:** adolescent, children, chronic disease, coronavirus, mental health, multimorbidity, pandemic

## Abstract

Despite the initial thrust of research aimed at understanding the impact of the COVID-19 pandemic on youth with physical illness and their parents, knowledge gaps in the literature remain, providing the impetus for additional investigation. This study described changes in psychological distress from prior to during the COVID-19 pandemic for parents and youth with physical illness, compared parent-proxy and youth self-reported perceptions of COVID-19-related psychosocial health, and modeled factors associated with psychological and psychosocial distress. There were 147 parent–youth dyads (2–16 years) from MY LIFE—a longitudinal study of youth with physical illness. The Kessler-6 (K6) measured psychological distress for the time before the COVID-19 lockdown (December 19 to March 20) and during the pandemic (December 20 to March 21) among parents and youth. COVID-19-related psychosocial health was measured using the CRISIS. Parents and youth reported increases in K6 scores (*d* = 0.62 and 0.38). Parent-proxy reports on the K6 were lower vs. youth self-reports prior to and during the pandemic (*d* = 0.63 for both). In contrast, parents reported lower proxy CRISIS scores for worries (*d* = 0.38) and effects of social restrictions (*d* = 0.52). Pandemic parent K6 scores were associated with age, combined in-person and online schooling for youth, COVID-19-related worries, and effects of social restrictions. For youth, only COVID-19-related worries and effects of social restrictions were associated with K6 scores. Parent worries were associated with youth sex, parental stress, family functioning, online and combination learning, and social restrictions. Parental depression and worries were associated with effects of social restrictions. Youth worries were associated with online and combination learning, and social restrictions. Youth disability, online learning, and worries were associated with effects of social restrictions. Few clinical factors are associated with COVID-19-related psychological and psychosocial distress. Instead, parent/family factors and youth learning environment have prominent roles in predicting outcomes and have implications for the health, education, and social services systems.

## Introduction

The COVID-19 pandemic has generated substantial individual and societal burden and has exposed systemic weaknesses across sectors working to address this global crisis. These burdens appear to differentially impact specific segments of the population, including children and youth with chronic physical illnesses (e.g., asthma, diabetes, epilepsy) and their families (1, 2). Evidence is mixed regarding elevated risk for and clinical presentation of COVID-19 for youth with vs. without physical illness (3, 4); however, more definitive are findings that youth with physical illness report they are more concerned about COVID-19 compared with their healthy peers (5, 6). This has sparked calls to action to prioritize the assessment of mental health among youth with physical illness (7) and especially during the COVID-19 pandemic, which has resulted in significant declines in health service use for youth, potentially perpetuating further deterioration of physical, mental, and psychosocial health (8).

Indeed, increased stress and declines in mental health associated with COVID-19 have been reported by parents and youth in the general population (9–11) and among those families who have a child with a physical or mental illness (6, 12–19). For instance, Hawke et al. aggregated general community and clinical samples of youth aged 14–28 years in Canada and found significant declines in mental health from prior to during the COVID-19 (10). This reported decline was large in magnitude (η^2^ = 0.31) and that the deterioration of mental health was more pronounced in the community sample of youth (η^2^ = 0.02). Durcan et al. reported elevated anxiety and depression during the pandemic among a Turkish sample of parents of youth aged 2–18 years with rheumatological conditions compared with parents of healthy youth (*d* = 0.28 and *d* = 0.34, respectively) (20). Youth with rheumatological conditions self-reported elevated symptoms of anxiety compared with their peers (*d* = 0.33). No significant differences in mental health were reported across the different types of rheumatological conditions, consistent with pre-pandemic studies that support non-categorical approaches (21, 22) to understanding the intersection between physical and mental illness in children and youth (23–27). Notably however, studies have showed that clinical factors such as illness duration, severity, or exacerbations during the pandemic have no or minimal association with COVID-19-related mental health among youth with physical illness (16, 20). Few studies have investigated psychosocial health (e.g., quality of life, hopefulness) among youth during the COVID-19 pandemic; available data, however, suggest that COVID-19 has negatively impacted psychosocial health among this population. For instance, a population-based study of youth aged 7–17 years in Germany showed that youth had a significant increase in self-reported low health-related quality of life from prior to during the pandemic (*d* = 0.73) and that this change in psychosocial health was similar for males and females; however, data on youth with physical illness was not reported (28).

Despite the initial thrust of research aimed at understanding the impact of the COVID-19 pandemic on youth with physical illness and their parents, knowledge gaps in the literature remain, providing the impetus for additional investigation. First, given the relative infancy of the extant literature examining the impact of COVID-19 on the mental health of youth with physical illness, most studies are descriptive in nature (12–14, 19). This initial work was crucial in estimating the burden on these youth and their families and the next step in the research agenda requires analytic studies that model predictors for changes in mental health from prior to during the COVID-19 pandemic. This information is needed for health professionals and policy makers to identify at-risk youth and families and provide the supportive resources to prevent or minimize declines in mental health during the ongoing pandemic. Second, there is a pressing need for studies examining COVID-19-related psychosocial health. Prediction of individual and population outcomes directly related to the countermeasures implemented by governments and public health agencies to curb transmission of COVID-19 (e.g., lockdown ordinances, school closures) can be used to inform public health strategies as we move forward into subsequent waves of the current pandemic and for future global crises that would minimize psychosocial distress. Third, there is a paucity of information with regard to differences in the perception of COVID-19-related psychological distress and psychosocial health between youth with physical illness and their parents. While evidence suggests that agreement between parents and youth with chronic illness on such constructs is modest (29–33), it is unknown if such findings generalize in the context of a pandemic. Understanding parent and youth perspectives is fundamental to tailoring family-centered care—the gold standard for pediatric health care—especially in the context of COVID-19 and the shift of services provision from in-person to virtual formats (34–37).

To address these knowledge gaps, this longitudinal study described changes in psychological distress prior to and during the COVID-19 pandemic in parents and their children with chronic physical illness, compared parent-proxy and youth-reported perceptions of psychological distress and perceptions of COVID-19-related psychosocial health, and modeled factors predictive of psychological distress and psychosocial health among parents and youth.

## Materials and Methods

### Design and Procedures

Data come from an on-going longitudinal study of a clinical sample of children with a chronic physical illness and their primary caregiving parent—Multimorbidity in Children and Youth Across the Life-course (MY LIFE). The design and cohort are described briefly, as the MY LIFE protocol is freely available (38, 39). Families were recruited from outpatient clinics at a pediatric hospital (Ontario, Canada) and baseline data collected from August 2017 until November 2019. Children were eligible if they were aged 2–16 years, diagnosed by a health professional with a physical illness, and had a parent who could understand English. Nurses introduced the study and invited families to meet with research staff, who further described MY LIFE and obtained permission to contact them at a later time. Research staff scheduled in-person data collection (hospital or home) through structured interviews and self-reported questionnaires, or in rare cases, data were collected using mail packages, in which case structured interviews were conducted by telephone. In March 2020, all data collection from self-reported questionnaires shifted to mail packages on account of the COVID-19 pandemic. Of the 263 families enrolled in MY LIFE, 188 (71.4%) were in active follow-up and eligible for the current COVID-19 sub-study. In total, 161 (85.6%) consented to participate in the sub-study and 147 (78.2%) completed data collection. Parent reports were collected for all children (*n* = 147) and youth ≥13 years (*n* = 48) also provided self-reports. For this sub-study, psychological distress and COVID-19-related psychosocial health data were collected between December 2020 and March 2021 *via* an online survey (Qualtrics). March 2021 was used as a cut-point for data collection as it represented the 1-year anniversary of the COVID-19 pandemic in Canada and coincidentally, the onset of a third wave of COVID-19 infections in the country (40). For measures that asked about pre-pandemic experiences, participants were instructed to report on experiences during the 3 months prior to lockdown ordinances (i.e., December 2019 to March 2020). All participants provided informed consent, and MY LIFE received ethical approval.

### Measures

Psychological distress in parents and youth was measured using the Kessler-6 (K6), a generic scale assessing the frequency in which individuals felt nervous, hopeless, restless/fidgety, depressed that nothing could cheer them up, everything was an effort, and worthless (41). Responses used a five-point scale ranging from 0 = “none of the time” to 4 = “all of the time” with total scores having a 24-point range. The K6 has robust psychometric properties in adult and youth populations (42, 43). Internal consistency was α = 0.90 for parent-self report, α = 0.92 for parent-proxy report, and α = 0.96 for youth-self report.

COVID-19-related psychosocial health was measured using the Coronavirus Health Impact Survey (CRISIS) (44). Items on the CRISIS are rated on five-point scale from 1 = “not at all” to 5 = “extremely” and in some instances are recoded such that higher scores on the CRISIS indicate more severe/frequent symptoms or behaviors. COVID-19-related worry was measured using five items that assess worry of infection, influence on physical and mental health, and time spent reading or talking about COVID-19. Internal consistency for the worry subscale was α = 0.83 for parent-self report, α = 0.85 for parent-proxy report, and α = 0.77 for youth-self report. The effect of COVID-19-related social restrictions was measured using eight items that asked about stress of restrictions and impacts on social relationships. Specifically, the questions were as follows: How stressful have the restrictions on leaving home been for you? Have your contacts with people outside of your home changed relative to before the COVID-19 crisis in your area? How much difficulty have you had following the recommendations for keeping away from close contact with people? Has the quality of the relationships between you and members of your family changed? How stressful have these changes in family contacts been for you? Has the quality of the relationships with your friends changed? How stressful have these changes in social contacts been for you? How much has cancellation of important events (such as vacation, parties, weddings, etc.) in your life been difficult for you? Internal consistency for the social restrictions subscale was α = 0.74 for parent-self report, α = 0.69 for parent-proxy report, and α = 0.71 for youth-self report. A single question on the CRISIS asked, “How hopeful are you that the COVID-19 crisis in your area will end soon?” Using the same five-point scale, higher scores on this item indicated more hope. Substance use during the COVID-19 pandemic was measured using five items that assessed frequency of use of alcohol, vaping products, tobacco products, cannabis, and hard drugs (e.g., opiates, heroin, cocaine, etc.). Internal consistency for the substance use subscale was α = 0.75 for parent-self report. Two items examined the extent to which parents perceived that the COVID-19 pandemic created financial problems and concerns about stability of living situation.

Chronic physical illness among youth was categorized according to the International Statistical Classification of Diseases and Related Health Problems (ICD)-10. The categories were rheumatological, respiratory, neurological, hematological, gastroenterological, endocrine, and dermatological. Comorbid mental illness in youth was measured at baseline using the parent-reported Mini International Neuropsychiatric Interview for Children and Adolescents (MINI-KID). This structured clinical diagnostic interview is aligned with the ICD-10 and Diagnostic and Statistical Manual of Mental Disorders (DSM)-5 and was administered by trained research staff (45). The MINI-KID has been shown to be valid and reliable in clinical and population samples of youth (46, 47). The most common mental illnesses present in childhood and adolescence (major depressive episode, generalized anxiety disorder, separation anxiety disorder, social and specific phobia, attention-deficit/hyperactivity disorder, oppositional defiant, and conduct disorder) were assessed (48).

Youth disability and functioning was measured using the 12-item parent-reported World Health Organization Disability Assessment Schedule (WHODAS) 2.0. It measures disability in the domains of cognition, mobility, self-care, getting along, life activities, and participation (49). Higher scores on the WHODAS 2.0 indicate more impairment across these domains. Its psychometric properties are sound in child and adolescent populations, including those with physical or mental illness (50, 51). Its internal consistency was α = 0.88 in this study sample.

Parent mental health and family environment were measured using parent-reported versions of the Center for Epidemiological Studies Depression Scale (CESD), Parental Stress Scale (PSS), and McMaster Family Assessment Device (FAD). The CESD is a 20-item measure of depressive symptomatology, which includes the domains of positive and negative affect, somatic symptoms, and interpersonal relations (52). Items are scored on a four-point scale (total ranges from 0 to 60), with higher scores indicating greater frequency and severity of depressive symptoms. The CESD is widely validated and its psychometric properties are strong in samples of adults, including parents of children with physical or mental illness (53, 54). The internal consistency was α = 0.92 in this sample. The PSS is an 18-item measure of stress resulting from parenting across the domains of parental rewards and satisfaction, stressors, and lack of control (55). Responses on the PSS are based on a five-point scale and the total score ranges from 18 to 90; higher scores indicate more symptoms of parental stress. It has been validated in parents whose children have physical or mental illness (55, 56) and its internal consistency was α = 0.86 in this sample. The General Functioning subscale of the FAD was used to measure family pathology and functioning (57). This 12-item scale includes the domains of problem solving, communication, behavior control, affective involvement and responsiveness, and roles. Responses are scored on a four-point scale with the total score ranging from 0 to 36. The FAD is coded such that higher scores indicate better family functioning. The FAD has demonstrated robust psychometric properties (57, 58) and its internal consistency in this sample was α = 0.87.

COVID-19 exposure was measured using the CRISIS. Items asked parents about exposure to individuals with a COVID-19 diagnosis within or outside of the family home. Parents were also asked if their children attended school fully in-person, fully online (i.e., remote learning), or a combination of both in-person and online. Sociodemographic factors were also collected and included parent and youth age and sex, parent educational attainment, and annual household income.

### Analysis

Paired *t* tests were used to compare psychological distress prior to and during the COVID-19 pandemic, as well as to estimate differences in parent-proxy and youth-self reports of psychological distress and COVID-19-related worry and social restrictions. COVID-19-related hope was compared using the McNemar test. For all comparisons, effect sizes were computed to estimate magnitudes of association (Cohen's d and Kendall's τ). Agreement between parent and youth reports were estimated using the intra-class correlation coefficient and κ. Multiple linear regression models were computed to examine the demographic, clinical, family, and COVID-19-related factors associated with parent and youth psychological distress and COVID-19-related psychosocial health. These models were based on parent-self and parent-proxy reports. For the psychological distress models, pre-pandemic K6 score was included as predictor; thus, these models represent change in psychological distress from before to during the COVID-19 pandemic. Because only 48 youth provided self-reports, regression models could not be reliably computed. Instead, bivariable correlations were reported for associated factors. All hypothesis tests were two-sided and α = 0.05. Data were analyzed using SPSS 21.

## Results

### Sample Characteristics

The mean ages of children and parents in this study were 9.3 (4.1) and 40.2 (5.7) years, respectively. While there was an approximately equal distribution of sex among children (52.4% males), the majority of parents were female, specifically the biological mother (89.0%). There were some heterogeneity in the proportion of physical illnesses; the most common were rheumatological conditions (28.6%), respiratory conditions (23.8%), and hematological conditions (17.0%). The least common type of physical illness was neurological (4.1%). One-third (34.0%) of children screened positive for a comorbid mental illness. Parents were typically partnered (married or common-law; 87.7%), completed postsecondary education (78.8%), and reported annual household incomes of ≥$90,000 (58.9%). Additional sample characteristics are shown in Table 1. In total, 29 parents reported an exposure to a COVID-19 diagnosis (19.7%). Five families reported a COVID-19 diagnosis within the household (3.4%). One child in the study had a COVID-19 diagnosis.

**Table 1:** Sample characteristics at baseline.

**Characteristic**	**Mean (SD)**	**Frequency (%)**
**Child**		
Age, years	9.3 (4.1)	
Male		77 (52.4)
Immigrant		12 (8.2)
Illness duration, years	5.1 (3.8)	
Illness severity, WHODAS 2.0	7.2 (7.3)	
Illness type		
Rheumatological		42 (28.6)
Respiratory		35 (23.8)
Neurological		6 (4.1)
Hematological		25 (17.0)
Gastroenterological		10 (6.8)
Endocrine		15 (10.2)
Dermatological		14 (9.5)
Any comorbid mental illness		50 (34.0)
Major depressive episode		10 (6.8)
Generalized anxiety disorder		13 (8.8)
Separation anxiety disorder		12 (8.2)
Social phobia		10 (6.8)
Specific phobia		13 (8.8)
Attention-deficit hyperactivity		20 (13.6)
Oppositional defiant disorder		5 (3.4)
Conduct disorder		3 (2.0)
**Parent**		
Age, years	40.2 (5.7)	
Female		130 (89.0)
Immigrant		24 (16.3)
Partnered		128 (87.7)
Postsecondary graduate		115 (78.8)
Household income ≥$90,000		86 (58.9)
Depressive symptoms, CESD	9.8 (9.0)	
Parental stress, PSS	36.7 (8.7)	
Family functioning, FAD	28.1 (5.4)	

*WHODAS 2.0, World Health Organization Disability Assessment Schedule; CESD, Center for Epidemiological Studies Depression Scale; PSS, Parental Stress Scale; FAD, McMaster Family Assessment Device*.

### Changes in Psychological Distress

As shown in Table 2, all informants (parent-self, parent-proxy, and youth-self) reported increased symptoms of psychological distress, as measured by the K6, from prior to during the COVID-19 pandemic. The increase was largest for parent-self reports (*d* = 0.62, *p* < 0.001), followed by parent-proxy reports on all children (*n* = 147; *d* = 0.39, *p* < 0.001). Among the subset of parent-proxies whose children provided self-reports, a similar-sized increase in K6 scores was found (*n* = 48; *d* = 0.30, *p* = 0.048). Youth self-reported an increase in K6 scores that also corresponded to a medium-sized effect (*d* = 0.38, *p* = 0.010).

**Table 2:** Changes in psychological distress (K6).

**Informant/Target**	***N***	**Pre-COVID-19**	**During COVID-19**	**d**	***p***
Parent self	147	4.0 (3.6)	6.7 (4.9)	0.62	<0.001
Parent-proxy	147	3.5 (3.4)	4.7 (4.1)	0.39	<0.001
Parent-proxy^a^	48	4.7 (4.7)	5.5 (4.8)	0.30	0.048
Youth self	48	6.6 (5.7)	8.9 (7.2)	0.38	0.010

a *These are the subgroup of parents who have a child who provided self-reported data*.

*K6, The Kessler-6 measured psychological distress for the time before COVID-19 lockdown (December 19 to March 20) and during the pandemic (December 20 to March 21) among parents and youth*.

### Comparisons Between Parent and Youth Reports

Parent-proxy reports on the K6 were significantly lower compared with youth self-reports prior to (*d* = 0.63, *p* = 0.012) and during the COVID-19 pandemic (*d* = 0.63, *p* = 0.001). Significant differences in parent-proxy and youth self-reports for COVID-19-related psychosocial health, as measured by the CRISIS were also found (Table 3). However, in contrast to the K6, parent-proxy reports were lower for worry (*d* = 0.38, *p* = 0.011) and effect of social restrictions (*d* = 0.52, *p* = 0.001). Parent-proxy reports also seemed to overestimate youth perceptions for hope that the COVID-19 pandemic will end soon (τ = 0.33, *p* = 0.007).

**Table 3:** Psychosocial health related to COVID-19 [Coronavirus Health Impact Survey (CRISIS)].

**Construct**	**Parent self**	**Parent-proxy**	**Parent-proxy^a^**	**Youth self**	**d/τ**
Worry	15.6 (4.2)	11.8 (4.2)	13.3 (3.5)	11.7 (3.7)	0.38
Social restrictions	22.7 (5.3)	23.5 (5.2)	23.6 (4.8)	21.1 (4.4)	0.52
Hope					0.33
Not at all/slightly	48 (32.7)	40 (27.2)	7 (14.6)	19 (39.6)	
Moderately	38 (25.9)	33 (22.4)	15 (31.3)	10 (20.8)	
Very/extremely	61 (41.4)	74 (50.3)	26 (54.2)	19 (39.6)	
Financial concerns	3.6 (1.7)	–	–	–	–
Substance use	9.0 (4.3)	–	–	–	–

*Data are mean (standard deviation), except for hope, which is reported as frequency (percent). Effects (Cohen's d or Kendall's τ) were based on the difference between parent-proxy and youth self-reports; effects were statistically significant at p < 0.05*.

a *These are the subgroup of parents who have a child who provided self-reported data*.

Agreement between parent-proxy and youth self-reports were generally low across all measures. For the K6, the ICC = 0.47 (0.22, 0.67) prior to the COVID-19 pandemic, and ICC = 0.43 (0.17, 0.63) during the pandemic. For the CRISIS, worry was ICC = 0.31 (0.03, 0.54), effect of social restrictions was ICC = 0.46 (0.20, 0.66), and hope was κ = 0.31 (0.12, 0.50).

### Factors Associated With Psychological and Psychosocial Distress

In regression models of parent-self and parent-proxy reported psychological distress, no clinical factors were associated with K6 scores during the COVID-19 pandemic (Table 4). For parent-self reports, elevated pre-pandemic psychological distress (*b* = 0.35), older parent age (*b* = 0.13), elevated parent depressive symptoms (*b* = 0.11), and more COVID-19-related worries and effect of social restrictions (*b* = 0.38 and *b* = 0.32) were associated with elevated psychological distress during the pandemic. In contrast, having children in combination in-person and online learning was associated with lower psychological distress (*b* = −2.02). Similar results were found in the parent-proxy model; however, parent depressive symptoms (*b* = 0.03) and combination learning (*b* = −0.61) were not significantly associated with youth psychological distress during the pandemic.

**Table 4:** Factors associated with parent and youth psychological distress (K6).

	**Parent (self-report)**		**Youth (parent-proxy)**	
**Factor**	**Semi-adjusted**	**Fully adjusted**	**Semi-adjusted**	**Fully adjusted**
Adjusted R_sq_	0.34	0.66	0.45	0.69
Pre-COVID-19 distress, K6*^a^*	**0.41 (0.12)**	**0.35 (0.09)**	**0.78 (0.09)**	**0.61 (0.07)**
**Demographics**				
Child age, years	−0.18 (0.11)	−0.09 (0.08)	−0.03 (0.08)	−0.03 (0.07)
Male child	0.33 (0.73)	−0.93 (0.55)	0.70 (0.55)	−0.35 (0.44)
Parent age, years	0.03 (0.07)	**0.13 (0.05)**	0.05 (0.06)	**0.09 (0.04)**
No postsecondary degree	1.53 (0.90)	−0.14 (0.69)	0.77 (0.68)	0.44 (0.53)
Income ≥$90,000	−0.22 (0.78)	−0.06 (0.60)	−0.62 (0.59)	−0.87 (0.46)
**Clinical**				
Illness duration	0.08 (0.11)	0.06 (0.08)	−0.02 (0.08)	−0.01 (0.06)
Disability, WHODAS	**0.12 (0.06)**	0.04 (0.04)	0.05 (0.05)	0.07 (0.04)
Comorbid mental illness	−0.72 (0.87)	−0.56 (0.65)	−0.34 (0.66)	−0.77 (0.51)
**Parent mental health**				
Depression, CESD	**0.14 (0.05)**	**0.11 (0.04)**	0.01 (0.04)	0.03 (0.03)
Parental stress, PSS	−0.01 (0.05)	0.03 (0.04)	−0.01 (0.03)	−0.01 (0.03)
Family functioning, FAD	−0.10 (0.07)	−0.10 (0.05)	−0.02 (0.05)	0.01 (0.04)
**COVID-19-related**				
COVID-19 exposure		−0.53 (0.64)		−0.50 (0.50)
Learning environment				
In-person		Reference		Reference
Online		−1.12 (0.60)		−0.84 (0.47)
Combination		**−2.02 (0.08)**		−0.61 (0.60)
Worries^a^		**0.38 (0.08)**		**0.14 (0.06)**
Social restrictions^a^		**0.32 (0.06)**		**0.34 (0.05)**
Financial concerns^a^		0.32 (0.18)		–
Substance use^a^		0.10 (0.06)		–
Hope^a^		−0.07 (0.20)		−0.18 (0.16)

*Data are presented as regression coefficient (standard error). Results in bold are statistically significant at p < 0.05. COVID-19-related financial concerns and substance use were not measured in parent-proxy reports and, thus, not included in the youth model*.

a *In the parent model, parents provided self-reported data on their own experiences for these variables, whereas in the youth model, parents provided proxy-reported data on the experiences of their child*.

Bivariable analyses showed that elevated youth-self reported psychological distress during the COVID-19 pandemic was associated with pre-pandemic psychological distress (*r* = 0.68), female sex (mean difference 5.3), more COVID-19-related worries (*r* = 0.52) and effect of social restrictions (*r* = 0.57), and less hope that the pandemic will end soon (*r* = −0.44). One clinical factor, illness severity (as measured by the WHODAS 2.0), was found to be associated with psychological distress (*r* = 0.33). All associations were significant at *p* < 0.05.

As shown in Table 5, elevated parent COVID-19-related worry was associated with having a male child (*b* = 1.25), more parental stress (*b* = 0.12), poorer family functioning (*b* = −0.11), online and combination learning (*b* = 1.39 and *b* = 1.88), elevated pandemic psychological distress (*b* = 0.40), and COVID-19-related social restrictions (*b* = 0.13), financial concerns (*b* = 0.46), and lower substance use (*b* = −0.19). Effects of COVID-19-related social restrictions were associated with elevated parental depressive symptoms (*b* = 0.17), pandemic psychological distress (*b* = 0.52), and COVID-related worry (*b* = 0.23), and substance use (*b* = 0.20).

**Table 5:** Factors associated with parent and youth COVID-19-related psychosocial outcomes (CRISIS).

	**Parent (self-report)**		**Youth (parent-proxy)**	
**Factor**	**Worries**	**Social restrictions**	**Worries**	**Social restrictions**
Adjusted R_sq_	0.47	0.41	0.19	0.43
**Demographics**				
Child age, years	−0.01 (0.09)	−0.11 (0.12)	−0.07 (0.11)	−0.12 (0.11)
Male child	**1.25 (0.58)**	1.41 (0.77)	1.17 (0.72)	**1.79 (0.75)**
Parent age, years	−0.09 (0.06)	−0.15 (0.08)	−0.10 (0.07)	−0.07 (0.07)
No post-secondary degree	1.36 (0.71)	0.91 (0.95)	**2.44 (0.87)**	−0.52 (0.94)
Income ≥$90,000	0.41 (0.62)	−0.65 (0.82)	−0.30 (0.75)	1.11 (0.78)
**Clinical**				
Illness duration	−0.03 (0.08)	0.13 (0.11)	−0.01 (0.10)	−0.01 (0.12)
Disability, WHODAS	−0.02 (0.05)	−0.01 (0.06)	0.03 (0.06)	**−0.13 (0.06)**
Comorbid mental illness	0.58 (0.68)	0.60 (0.90)	−0.28 (0.83)	1.03 (0.87)
**Parent mental health**				
Depression, CESD	0.01 (0.04)	**0.17 (0.05)**	0.07 (0.05)	**0.11 (0.05)**
Parental stress, PSS	**0.12 (0.04)**	0.02 (0.05)	−0.08 (0.04)	0.05 (0.05)
Family functioning, FAD	**−0.11 (0.06)**	0.06 (0.07)	0.04 (0.07)	−0.06 (0.07)
**COVID-19-related**				
COVID-19 exposure	0.71 (0.67)	0.21 (0.89)	0.65 (0.83)	0.82 (0.88)
Learning environment				
In-person	Reference	Reference	Reference	Reference
Online	**1.39 (0.63)**	1.29 (0.84)	**1.62 (0.77)**	**1.84 (0.81)**
Combination	**1.88 (0.83)**	2.07 (1.10)	**2.15 (0.98)**	1.41 (1.04)
COVID-19 Distress, K6^a^	**0.40 (0.08)**	**0.52 (0.12)**	0.10 (0.11)	**0.81 (0.09)**
Worries^a^	–	**0.23 (0.12)**	–	**0.19 (0.09)**
Social restrictions^a^	**0.13 (0.07)**	–	**0.17 (0.08)**	–
Financial concerns^a^	**0.46 (0.19)**	0.03 (0.26)	–	–
Substance use^a^	**−0.19 (0.07)**	**0.20 (0.09)**	–	–
Hope^a^	−0.08 (0.21)	−0.51 (0.27)	−0.07 (0.26)	0.39 (0.27)

*Data are presented as regression coefficient (standard error). Results in bold are statistically significant at p < 0.05. COVID-19-related financial concerns and substance use were not measured in parent-proxy reports and thus, not included in the youth model*.

a *In the parent model, parents provided self-reported data on their own experiences for these variables, whereas in the youth model, parents provided proxy-reported data on the experiences of their child*.

In the parent-proxy model, youth COVID-19-related worry was associated with lower parental education (*b* = 2.44), online and combination learning (*b* = 1.62 and *b* = 2.15), and social restrictions (*b* = 0.17). For the COVID-19-related social restrictions model, associated factors were male sex (1.79), lower illness severity (*b* = −0.13), parental depressive symptoms (*b* = 0.11), online learning (*b* = 1.84), elevated pandemic psychological distress (*b* = 0.81), and COVID-19-related worry (*b* = 0.19). According to youth-self reports, COVID-19-related worry and social restrictions were correlated (*r* = 0.50), and females reported higher scores on these CRISIS scales (mean difference 3.5 and 2.7, respectively).

## Discussion

### Summary of Findings

In this clinical sample of youth with chronic physical illness and their parents, levels of psychological distress increased from before to during the COVID-19 pandemic. Parent-proxy reports appear to underestimate psychological and psychosocial distress in youth, but overestimate youth perceptions of hope that the pandemic will end soon. Furthermore, clinical factors were generally not found to be associated with psychological or psychosocial distress; instead, parent mental health, COVID-19-related stressors, and learning environments that were online only or a combination of in-person/online were associated with parent and youth psychological and psychosocial distress during the pandemic.

Consistent with previous studies, parents of children with physical illness reported a medium-sized increase in psychological distress from prior to during the COVID-19 pandemic (13, 16, 19, 20, 34). This deterioration in the mental health of parents has pervasive sequelae, negatively affecting their well-being (59), and also the health and well-being of their children (60–62). Psychological distress has been shown to hinder caregiving responsibilities, which can result in poorer health outcomes for children with physical illness (63–65), many of which have complex care needs that may be compounded in the context of the COVID-19 pandemic. Intuitively, higher baseline depressive symptoms and current COVID-19-related worry and effects of social restrictions were associated with larger increases in psychological distress; these are unique, but related constructs that capture overlapping internalizing psychopathologies (66). In contrast, having children in combined in-person and online learning was associated with lower psychological distress during the pandemic. Evidence is mounting that parental burden during the COVID-19 pandemic stems, in large part, from having to support their children with at-home or remote online learning, while schools are closed (67–69). Combined in-person and online learning may afford parents a bit of relief of the burden of managing their children's learning during in-person schooling (68), and also provide a sense of control in their families' schedules when children are involved in online learning. This notion is speculative and further research—quantitative and qualitative—is needed to substantiate or refute this hypothesis as previous work has suggested that parents have reported feeling less supported by schools and having limited contact with teachers during temporary periods of online learning (69).

Youth also experienced an increase in psychological distress, as reported by parent-proxies and themselves, though this change was relatively smaller compared with the change in psychological distress experienced by parents. This finding was consistent with previous studies in children and adolescents in the general population (6, 9, 11), as well as among youth with physical or mental illness (12, 15, 17, 28). While the increase in psychological distress was seen over a relatively short follow-up, evidence suggests that psychopathology in youth with physical illness (i.e., physical-mental comorbidity) is chronic in nature (26) and results in compounding negative effects on youth health and well-being (23). Given that this comorbidity appears early in life, there is the potential for poor health outcomes over the life-course and increased burden on the health system resulting from the often complex care needs of youth with physical–mental comorbidity (27, 70, 71).

While both parent and youth reports of elevated COVID-19-related worry and social restrictions were associated with increased psychological distress during the pandemic (consistent with previous studies) (16), only for youth was the association between low hope and elevated psychological distress found. This finding, consistent with previous research, offers a potential target for intervention to prevent declines in youth mental health, foster adaptive coping and resilience, and promote positive psychology during times of population crisis (72, 73). In collaboration with public health and social services, the education system is well-positioned to deliver universal preventive interventions for youth. By incorporating prevention proactively within curricula developmentally appropriate and evidence-based interventions can be applied, stigma associated with help-seeking for mental health services can be reduced, and better support be provided to vulnerable youth (74–77).

Among youth reports, the finding that females had elevated psychological distress was consistent with previous work highlighting sex differences related to internalizing symptoms (48, 78). Also, the finding that disability was associated with increased psychological distress during the pandemic reinforces the negative consequences of social restrictions for youth with physical illness and the interrelationship between disability, physical illness, and mental health among youth (50).

Family-level factors were implicated in psychosocial outcomes related to the pandemic; families reporting more stressful environments and worse functioning tended to have more worry about COVID-19. Financial concerns were also associated with COVID-19-related worry and likely the stress from such hardship, in combination with stressful parents and family dysfunction, are compounded in families who have a child with a physical illness or complex care needs (19, 68). Further, parent substance use was associated with lower levels of worry, but higher levels of the effect of social restrictions may suggest that parents are using substances to self-medicate their anxiety surrounding COVID-19 (79), as well as engage in negative coping behaviors in response to burn-out (28, 80, 81). In sum, these findings speak to the importance of adopting and refining family-centered models of care that integrate physical and mental health specialties for children with physical illness and their families, especially during times of global crisis (16, 36, 37, 82).

In contrast to the inverse association with psychological distress, online and combination learning environment was positively associated with COVID-19-related worry among parents. Such worry may result from parental concerns regarding muted educational progress and fewer future opportunities for children, especially those achieving academic milestones (e.g., beginning secondary or postsecondary school). These learning environments may also serve as constant reminders for parents that life has deviated from normalcy (e.g., switching between in-person and online learning due to transmission outbreaks) leading to rumination, a core symptom of anxiety disorder (66), on the pandemic. Ultimately, the compounding stressors of caregiving to their children with physical illness, supporting their education, and often managing careers—all from home—contribute to burn-out and, a general sense of loss among parents (28, 67, 79, 80, 83, 84).

Based on parent-proxy reports, lower level of disability was associated with higher level of negative effects of COVID-19-related social restriction among youth with physical illness. This finding may be a function of a perceived sense of loss among youth who, in the context of their physical illness (85–87), maintain a relatively high level of functioning or minimal impairment. For these youth, the shutdown of organized sports, closure of parks and recreational facilities, as well as other closures in response to the COVID-19 pandemic essentially removed opportunities for physical activity and social interaction with peers (67, 69). The loss of such activities, which in the context of school closures, provided some routine for youth with physical illness and their families likely contributed to their reports of the effects of social restriction, requires additional study. In a related vein, online and combination learning were associated with parent-proxy reports of COVID-19-related worry and social restrictions among young. There is robust evidence that these learning environments, which reduce social interactions among youth (88, 89), may also contribute negatively to their mental health—an effect that may be compounded in youth with physical illness who may already experience individual and family burden (67, 68, 90–92).

Of interest is the discrepant finding between informants with regard to the association between youth sex and COVID-19-related social restrictions. For youth-self report, female sex was associated with increased perception of the negative effect of social restrictions, whereas for parent-proxy reports, this association was with male sex. This informant inconsistency may be related to differences in the interpretation of sex as a biological construct vs. gender as a sociocultural construct. Additional research, likely qualitative in nature, is needed to investigate this notion; however, this finding confirms the importance of including multiple informants and perspectives in the context of child and family research.

### Study Limitations

There are some notable limitations to this study. First, recall bias may affect the validity of the findings as parents and youth were asked to retrospectively reflect on their psychological and psychosocial health prior to the COVID-19 lockdown ordinances. Thus, change in distress scores may be overestimated. Ongoing data collection in MY LIFE may permit the examination of post-pandemic psychological and psychosocial health in a prospective manner. Second, the absence of a control group prevents ascertainment as to whether the changes in psychological distress and psychosocial health are specific to youth with physical illness and their families, or whether the magnitude of such changes are similar to subgroups within the population. Given that few clinical factors were associated with psychological or psychosocial distress, it is reasonable to expect similar findings in the general population. Third, because a relatively small subsample of youth was age eligible to provide self-reports, this study was underpowered to reliably estimate measures of association in adjusted models of psychological distress and psychosocial health.

## Conclusion

Youth with chronic physical illness and their parents report substantial increases in symptoms of psychological distress from before to during the COVID-19 pandemic. Both also report elevated COVID-19-related worry and negative effects of social restrictions aimed at reducing transmission. Few clinical factors were associated with changes in psychological distress or COVID-19-related psychosocial health; instead parent/family factors and online or combined online and in-person learning environments were key risk factors in predicting poorer outcomes in this vulnerable population of youth and their families. Models of care that are family-centered are crucial to support the mental and psychosocial health of youth with physical illness and their families, especially during times of global crisis. Efforts within the health system to integrate physical and mental health care, as well as trans-sectoral efforts across the health, education, and social services systems must be redoubled to capture the value of youth perspectives to provide learning environments that accommodate the unique needs of youth with physical illness and their parents to reduce burden and promote the best possible health outcomes.

## Data Availability Statement

The datasets presented in this article are not readily available because the authors do not have ethical approval to share the study data. Requests to access the datasets should be directed to Mark A. Ferro, mark.ferro@uwaterloo.ca.

## Ethics Statement

The studies involving human participants were reviewed and approved by Waterloo Human Research Ethics Board. Written informed consent to participate in this study was provided by the participants' legal guardian/next of kin.

## Author Contributions

MF conceptualized the study, led the acquisition of funding, supervised all aspects of data collection, led the analysis of the data, and drafted the manuscript. SM, JY, SR, EL, and JG contributed to study design, acquisition of funding, provided insight to the interpretation of findings, and edited the manuscript for intellectual content. All authors agree to be accountable for the content of the work.

## Funding

This study was funded by grants from the Canadian Institutes of Health Research (PJT-148602 and MS1-173067). MF holds the Canada Research Chair in Youth Mental Health and is the recipient of the Early Researcher Award from the Ontario Ministry of Research, Innovation and Science.

## Conflict of Interest

The authors declare that the research was conducted in the absence of any commercial or financial relationships that could be construed as a potential conflict of interest.

## Publisher's Note

All claims expressed in this article are solely those of the authors and do not necessarily represent those of their affiliated organizations, or those of the publisher, the editors and the reviewers. Any product that may be evaluated in this article, or claim that may be made by its manufacturer, is not guaranteed or endorsed by the publisher.
